# Socially Embedded Digital Coaching Platform to Promote Healthy Lifestyle and Mental Health in Underprivileged (Pre)Pregnant Couples and Young Families (Our Smart Family Buddy Program): Protocol for a Mixed Methods Study

**DOI:** 10.2196/88913

**Published:** 2026-10-15

**Authors:** Teuntje Wieles, Hilmar Bijma, Ageeth Rosman, Ineke Bolt, Marina Bos-de Vos, Isabelle Fabbricotti, Annelli Janssen, Koen Joosten, Evangelos Niforatos, Valentijn Visch, Régine Steegers-Theunissen

**Affiliations:** 1 Department of Obstetrics & Gynecology, Erasmus MC, Dr Molewaterplein 40, Rotterdam, The Netherlands, 31 0107040704; 2 Department of Care Ethics, University of Humanistic Studies, Utrecht, The Netherlands; 3 Innovation Center of Care, Rotterdam University of Applied Sciences, Rotterdam, South Holland, The Netherlands; 4 Section Medical Ethics, Philosophy and History of Medicine, Department of Public Health, Erasmus MC, Rotterdam, The Netherlands; 5 Faculty of Industrial Design Engineering, Delft University of Technology, Delft, The Netherlands; 6 Department of Health Services Management and Organization, Erasmus School of Health Policy & Management, Erasmus University Rotterdam, Rotterdam, The Netherlands; 7 The Dutch Research Institute for Transitions (DRIFT), Erasmus University Rotterdam, Rotterdam, South Holland, The Netherlands; 8 Department of Neonatal and Pediatric Intensive Care, Erasmus MC, Rotterdam, The Netherlands; 9 Sophia's Children Hospital, Erasmus MC, , Rotterdam, Netherlands

**Keywords:** periconception health, digital lifestyle platform, cocreation, vulnerable families, deprived neighborhood

## Abstract

**Background:**

The early life course, that is, from preconception up until the first 1000 days of life, is pivotal for optimizing parental and child health. In deprived settings, unfavorable lifestyle behaviors and poor mental health are more prevalent, while access to effective, context-sensitive support is limited. Digital interventions combined with community-based approaches may help address these disparities.

**Objective:**

This protocol describes the methodology and initial progress of the Our Smart Family Buddy (OSFB) program, which aims to develop, implement, and evaluate a socially embedded digital coaching platform to improve nutrition and mental well-being among (pre)pregnant couples and families in deprived neighborhoods.

**Methods:**

The OSFB program is a multiphase mixed methods program conducted within a transdisciplinary consortium consisting of 6 interrelated work packages. The program is structured in 3 phases: phase 1, exploration and preparation; phase 2, cocreation, design, and development; and phase 3, prototype evaluation and optimization. Data collection includes quantitative questionnaires, qualitative interviews and focus groups, and digital user data from the platform. The primary outcomes include lifestyle behaviors (nutrition and sleep), mental well-being, user engagement, and implementation outcomes, including feasibility, acceptability, and reach.

**Results:**

The program was funded in 2022. A transdisciplinary consortium was established in 2023. Data collection and analysis for phase 1 were conducted between 2023 and 2025, including literature reviews and exploratory interviews. A deprived neighborhood in the municipality of Rotterdam, the Netherlands, was selected as the target neighborhood. The target population was defined as (pre)pregnant couples and families with a child aged <4 years. Phase 2 is ongoing (2024‐2026), focusing on iterative design through locally established field labs and community building. Phase 3 is planned for 2026 to 2028.

**Conclusions:**

This protocol describes a socially embedded, multiphase approach to developing and implementing a digital coaching intervention for families in vulnerable circumstances. Through a transdisciplinary approach within a real-world, local community setting, the OSFB program aims to develop a context-sensitive intervention to support sustainable behavior change in early life.

## Introduction

The early life course—from at least 100 days before conception up until 1000 days after conception—is the most critical period in life, shaping long-term health outcomes [1,2]. During this time span, unfavorable (parental) lifestyle behaviors, including poor nutrition, smoking, alcohol use, and physical inactivity, combined with mental stress and unfavorable living conditions, increase the risk of complications before and during pregnancy and childhood, resulting in impaired health during the life course of both children and parents [3,4]. In deprived neighborhoods, these risks are further increased by the co-occurrence of multiple adversities resulting from socioeconomic disparities, such as poor housing, financial insecurity, language barriers, unemployment, and limited access to support and care. Furthermore, limited access to supportive relationships from family and community networks impedes resilience and interacts with unfavorable lifestyle and stress [5]. Together, these conditions leave families in a near-constant state of alert or “survival mode,” reducing cognitive capacity and self-efficacy—both of which are essential for engaging with and benefiting from preventive lifestyle programs [6]. Furthermore, families in deprived neighborhoods often encounter barriers in accessing health-promoting resources because of physical distance, financial constraints, limited services, or limited health literacy [5]. Unfortunately, current health services are poorly tailored to the needs of underprivileged families. The burden of adversity and lack of resources are reflected in the life expectancy of families living in deprived neighborhoods being 5 years shorter, with up to 15 to 20 more years in poor health compared to families in more prosperous neighborhoods [7,8]. Next to the individual impact, these disparities also incur health care and societal costs, as the burden of chronic diseases and reduced labor participation strains both the health system and the broader economy [9].

To decrease these inequities and prevent their transgenerational transmission, it is crucial to focus on improving lifestyle and interrelated mental health of (pre)pregnant couples and young families [10]. Digital lifestyle interventions offer promising advantages: they are personal, scalable, and flexible in time and place and can engage the target population through technology [11-13]. However, as community involvement is often neglected in development, digital tools frequently lack alignment with users’ lived experiences, values, and digital competencies and, as a result, fail to reach people in deprived neighborhoods [14]. Additionally, such tools may insufficiently take into account system-level disadvantages and users’ mistrust, particularly toward health care systems, which may undermine their accessibility, uptake, and effectiveness [15,16]. To sustainably reduce health inequities, digital lifestyle interventions should be complemented by broader structural and systemic efforts that address also underlying social and environmental determinants of health [17]. When digital tools are cocreated with the target population and embedded within supportive local networks and communities, they may contribute to providing accessible and scalable support.

From this background, we launched the Our Smart Family Buddy (OSFB) program, funded by the Dutch Research Council (Kennis & Innovatie Convenant Lifestyle and Living Environment program). Our primary aim is to develop a socially embedded digital platform to improve nutrition and reduce stress in (pre)pregnant couples and young families living in a deprived neighborhood in Rotterdam, the Netherlands. To achieve this, the social part and functionalities of the digital Family Buddy platform, as part of the OSFB program, will be cocreated with the target population and involved stakeholders and embedded within their living environment, local communities, and networks. The Family Buddy platform will be flexible, trustful, and tailored to the individual context and needs—through personalized lifestyle coaching—and the systemic and social level by fostering social support connections, including trust building, social cohesion promotion, and disseminating best practices to help the target populations navigate systemic stressors. The main research question is whether a cocreated, tailored, interactive socially embedded digital platform using a life course approach can support self-management of coping with stress and adopting sustainable lifestyle changes. This paper describes the insights of the first 3 years of the OSFB program and the applied methodology for developing the social environment and the Family Buddy platform.

## Methods

### OSFB Consortium

To effectively address the complex factors influencing (digital) lifestyle behavior change, the OSFB program follows a quadruple helix model, bringing together key stakeholders from 4 domains: society (eg, (pre)pregnant couples, networks, and community organizations), academia (eg, researchers in public health and digital innovation), industry (eg, app developers and health tech companies), and government (eg, local municipalities and public health services) [18]. These stakeholders are selected based on their experience, knowledge, and expertise and collaborate in 6 interrelated work packages (WPs). Each WP is composed of several experts from different organizations and contributes to a specific aspect of the development of the social environment and the Family Buddy platform prototype, further referred to as the Family Buddy.

### Program Overview

The project was funded in 2022. The planned duration of the OSFB program is 6 years, divided into 3 (semioverlapping) phases, as illustrated in Figure 1: phase 1, preparation and exploration (2022‐2025); phase 2, cocreation, design, and technological development (2024‐2026); and phase 3, prototype evaluation and optimization (2026‐2028).

**Figure 1.**
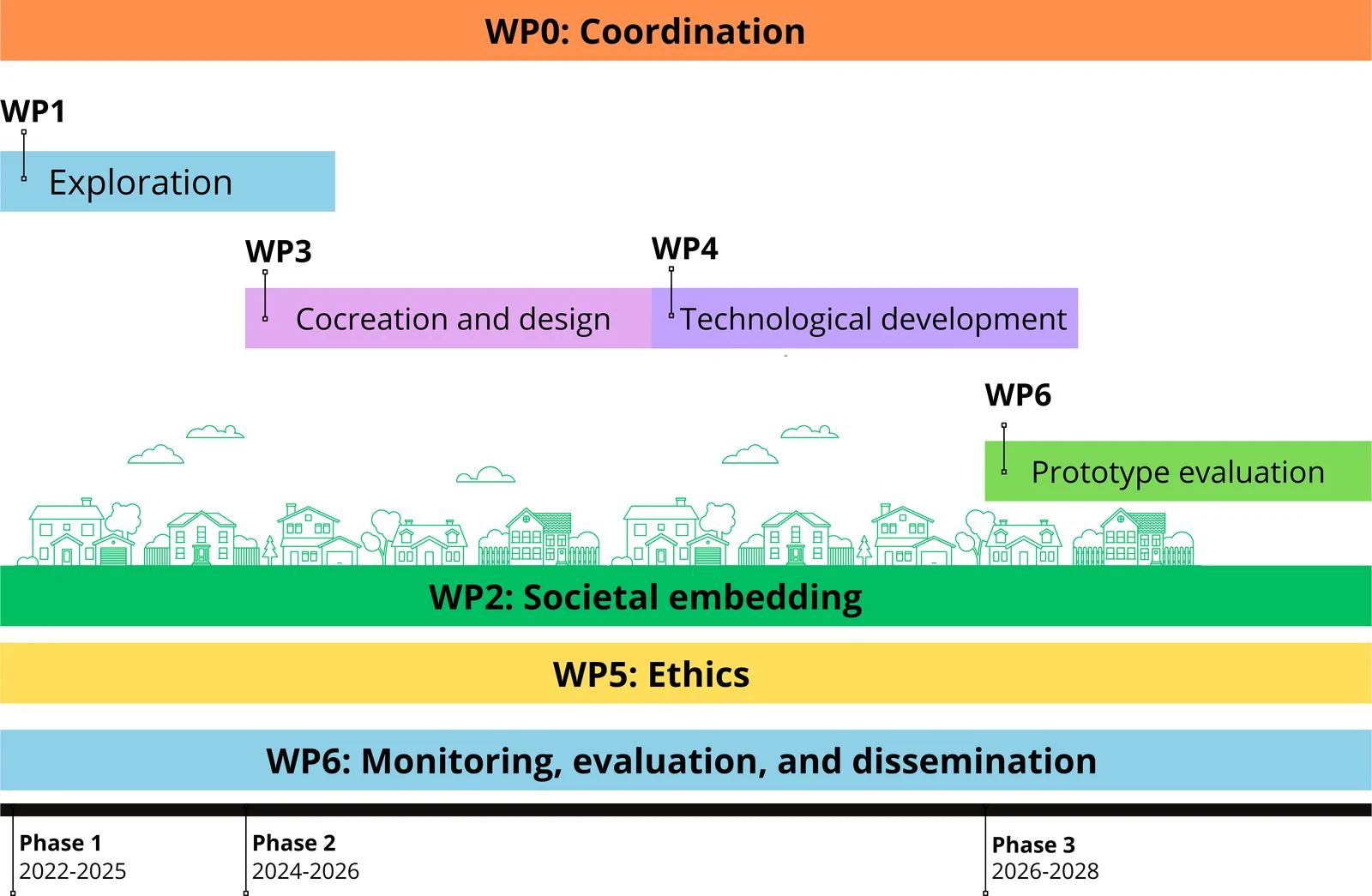
Visual representation of the 3 overlapping phases of the program and the involvement of the work packages (WPs) per phase.

These 3 phases, guided by the program’s coordination WP (WP0) and supported by the societal embedding WP (WP2) and ethics WP (WP5), are described in more detail in the following section.

### Coordination and Organization

#### Overview

The coordination and organization of the program is carried out by WP0. This WP includes a management team composed of the principal investigator, project coordinator, and project manager. The management team organizes the regular consortium and core team meetings and is responsible for the methodology used to build the quadruple-helix consortium and the launch of the Family Buddy. WP0 reports the yearly proceedings of the content and finances of the OSFB program to the Dutch Research Council (NWO).

#### Phase 1: Exploration and Preparation (Years 1 and 2)

In the first phase of the program, the consortium is established, and the WPs are defined. The activities in this phase are essential for building the consortium, strengthening societal embedding, and ensuring ethical monitoring. The responsibility of WP1 is to identify which functionalities of (existing) eHealth tools are most suitable for integration into the Family Buddy. Therefore, WP1 assesses the availability and relevance of existing eHealth tools for promoting healthy lifestyles for (future) parents and families in deprived circumstances using scoping reviews and semistructured qualitative interviews. For the literature reviews, the search strategies are developed with a medical librarian and include key terms related to the aim of the studies. Screening of the literature is realized using ASReview (version 1.1; developed by Utrecht University). For the interviews, coding is primarily driven by the research questions, aimed at identifying barriers and facilitators to eHealth use, as well as opinions on eHealth features. ATLAS.ti (Lumivero, LLC) was used to code the transcripts. A thematic analysis following Braun and Clarke is conducted, incorporating elements of framework analysis. Identified barriers and facilitators to eHealth use are indexed according to the COM-B (capability, opportunity, motivation, behavior) model and the Theoretical Domains Framework [19-21].

Furthermore, best practices for implementation strategies are identified by WP1 during this phase based on a systematic review of the existing literature [22-24]. The outcomes of phase 1 guide the content and structure of the Family Buddy, as well as its implementation and dissemination plan.

#### Phase 2: Cocreation, Design, and Technological Development (Years 2-4)

Building on the insights from phase 1, phase 2 focuses on cocreating the Family Buddy with the target population and stakeholders in the neighborhood as social environment. Using findings from interviews and cocreation sessions, a concept Family Buddy will be developed.

WP3 conducts semistructured interviews using interview guides (Supplementary info 1 in Multimedia Appendix 1) to obtain a deeper understanding of the values, value tensions, and challenges families in deprived circumstances face when attempting lifestyle changes. Sensitizing exercises and probe materials (ie, cards and visuals of the emerging prototype) will allow participants to more easily discuss difficult moments they experience when making lifestyle choices and coping with stress, as well as their preferences for how to deal with such difficult moments using a digital buddy. In parallel, focus groups and interviews with health care professionals explore how the Family Buddy could be integrated into current practices, identifying facilitators and barriers, and perceived added value. This input directly informs the content and design specifications of the Buddy from a health care perspective.

WP3 also coordinates cocreation sessions with other WPs and project partners within the consortium to narrow down the various ideas and to translate insights from all WPs into concrete design choices for the Family Buddy. To ensure that the Family Buddy aligns with the needs of the target population, cocreation sessions are conducted within the locally established field labs and public spaces, such as playgrounds. These sessions make use of a session guide and probes, including visuals of the emerging Family Buddy. The sessions are integrated into community activities and involve local stakeholders to enhance social cohesion and foster a sense of community ownership of the program and Family Buddy.

The content of the digital part of the Family Buddy is based on experience within our consortium with 4 different programs: Smarter Pregnancy, Pregnant Faster, Smarter Eating with your Child, and FlyKIDS. Smarter Pregnancy [25,26] is a digital lifestyle coaching program previously developed within the Erasmus MC University Medical Center and implemented in existing health care practices. On the basis of the Smarter Pregnancy, the preconception lifestyle tool Pregnant Faster is created, in cocreation with women in underprivileged circumstances [27,28]. This program, supporting lifestyle in the preconception phase, is further developed and implemented in the Family Buddy. Its implementation serves as a valuable opportunity to generate insights into user engagement, evaluation methods, and strategies for integrating digital tools into routine care. FlyKIDS, a lifestyle screening tool to be used in youth health care (FlyKIDS), is developed and will be further implemented in the Family Buddy [29].

Our knowledge and experience with these 4 programs, together with the output from the reviews in phase 1 and the cocreation sessions and interviews in phase 2, will establish the framework for the socially embedded digital Family Buddy. In addition, Family Buddy will include AI-based functionalities combining applied research with technology design, provided by WP4. Central to this phase is the development of large language model (LLM)–based conversational tools within WP4 that support families in self-driven learning and behavior change related to lifestyle. LLM tools are developed using Retrieval-Augmented Generation architecture, which embeds context into prompts to improve agent performance. Context is retrieved from public documents and guidelines created by national institutions, including the Center for Youth and Families and National Youth Center. Expected key performance indicators characterizing a healthy lifestyle for young (future) parents and their children are quality of nutrition and reduction of stress. The LLM-based conversational tools will be informed by existing, validated information sources, including national and regional guidelines.

#### Phase 3: Prototype Evaluation and Optimization

Although evaluation, implementation, and dissemination activities are embedded throughout phases 1 and 2, phase 3 (WP6) focuses on the formal evaluation and iterative optimization of the Family Buddy prototype toward a minimal viable product. The primary objective of this phase is to assess real-world engagement, usability, and short-term effectiveness of the Family Buddy among (future) families in vulnerable circumstances using a mixed-methods pilot study design.

For the quantitative component, which includes questionnaires and app analytics, a target sample size of approximately 50 participants is deemed appropriate. Methodological guidance for pilot studies indicates that samples in the range of 30 to 50 participants are generally sufficient to estimate key parameters, assess feasibility, and inform the design of larger studies [30-32]. Quantitative data will be collected through validated questionnaires and app analytics over a study period of 2 months per participant. The primary outcomes include changes in lifestyle behaviors (eg, nutrition, physical activity, smoking, alcohol use, and stress management), changes in mental health (including self-image, mood, and daily functioning), and actual usage of the Family Buddy. The secondary outcomes focus on user interaction patterns, such as engagement with specific modules and features, frequency of active days, and login behavior, assessed through log data (eg, via Google Analytics).

The qualitative component will involve approximately 15 to 24 participants and aims to provide in-depth insight into how, why, and under which circumstances the Family Buddy is used, how it integrates into daily life, and how these experiences relate to lifestyle change [33]. Data will be collected through a combination of questionnaires, in-depth interviews on lifestyle changes, concept mapping, Q-methodology, and continuous log data monitoring. Two complementary data collection tracks will be applied: one focusing on baseline and post-intervention questionnaires combined with interviews and continuous log data collection and another incorporating context mapping at 2 to 3 weeks and Q-methodology at the end of the study period. Usability will additionally be assessed using validated instruments, including the mHealth App Usability Questionnaire [34]. This validated questionnaire, developed by Zhou et al, assesses the usability of mobile health apps, consisting of 18 to 21 Likert-scale items, covering perceived usefulness, ease of use, and satisfaction.

Following this pilot phase, findings will be used to optimize the Family Buddy toward a minimal viable product. Subsequently, an observational cohort study will be conducted to evaluate the improved intervention in a real-world setting, with a stronger focus on implementation processes and behavioral change mechanisms. As the intervention and its context are expected to evolve based on insights from earlier phases, the exact study design, outcomes, and intermediate measures for this subsequent phase will be further refined. This approach ensures a flexible and iterative evaluation process, allowing the Family Buddy to be continuously adapted to the needs and realities of the target population.

### Societal Embedding

The societal embedding of the program and the social part of the Family Buddy is facilitated by WP2. This is established through leveraging existing resources and networks of organizations and communities of individuals within the neighborhood in 4 field labs [35]. In phase 1, WP2 focuses on the development of field lab methodology and guidelines based on existing literature using a scoping review. In phase 2, 4 field labs are established, focusing on different aspects of a healthy living environment: food, movement, mental or social health, and health care providers. These field labs serve as cocreation environments, where stakeholders, including the target population, local health care providers, and community organizations, cocreate a vision and insights on the barriers and possible actions in a transformation toward a healthier living environment in the neighborhood. The networks strengthened in these field labs facilitate the societal embedding of the Family Buddy and facilitate its cocreation, testing, and implementation. The research outcomes of WP2 include insights into community needs, community dynamics, and transformative potential of the neighborhood to create a healthier and supportive living environment to facilitate the dissemination and implementation of the Family Buddy in phase 3 through societal embedding in the neighborhood.

### Ethical Considerations

The OSFB program comprises multiple studies conducted across different participating institutions and universities. For all studies, ethical approval has been obtained and will be obtained separately for each study in accordance with institutional policies. For each study involving human participants, ethical approval is obtained from the relevant institutional review board or medical ethics committee of the participating institution(s), that is, Erasmus University Medical Center Medical Ethical Committee and TU Delft Human Research Ethical Committee. Approval details, including committee names and application or reference numbers, are reported in the respective individual publications and in the overview of the conducted OSFB studies within each of the WPs.

For all studies involving primary data collection, written informed consent is obtained from all participants prior to participation, in line with Medical Research Ethics Committee requirements. For studies using secondary or existing data, ethical approval and consent procedures are covered within the original study protocols in accordance with applicable regulations.

Regarding privacy and confidentiality, all data are handled in accordance with the General Data Protection Regulation and national data protection laws. Data are anonymized or pseudonymized prior to analysis and determined at the level of individual studies within the program. Access to identifiable data is restricted to authorized consortium members only.

Participant compensation is also determined at the level of individual studies within the program and approved by the ethics committees. Details regarding data collection and participant compensation are described in the respective research papers.

Additionally, the ethical challenges of developing and implementing the Family Buddy are addressed by WP5. In phase 1, an ethical framework will be developed based on the findings of a narrative review. This review examines existing ethical frameworks for public health, lifestyle interventions, and digital lifestyle interventions. The resulting ethical framework will facilitate the development of the Family Buddy in phases 2 and 3, adhering to ethical principles, among which are justice, privacy, and inclusivity [24]. Furthermore, WP5 provides guidance on the moral acceptability of the use of possible manipulative influences (eg, rewards) through an ethical analysis of the findings of an interview study with potential users of the Family Buddy (see Supplementary info 2 for interview guide in Multimedia Appendix 1). WP5 is also responsible for ethical monitoring during all 3 project phases to identify ethical concerns of OSFB users regarding the responsible use of AI and the development and long-term deployment of the Family Buddy with particular attention to issues of justice and health inequity.

### Monitoring, Evaluation, and Dissemination

Monitoring, evaluation, and dissemination activities are integrated throughout all phases of the program to ensure continuous learning and adaptation to emerging insights from the target population and other stakeholders. As the program progresses, the insights collected from cocreation sessions, interviews, and literature reviews will inform ongoing design decisions and guide the development process. An iterative monitoring approach enables timely adjustments to keep the Family Buddy aligned with the needs and values of the target population and with the societal context.

In parallel, dissemination is treated as an ongoing process, with regular program updates shared with stakeholders and the community, to maintain engagement and foster a sense of ownership. Research findings, program progressions, and recruitment campaigns are shared through presentations and consortium meetings, as well as through local key persons in the neighborhood. As the program progresses, more formal dissemination pathways, including scientific publications and conference presentations, will be used to share outcomes and insights with a broader academic and professional audience.

### Stakeholder Selection and Recruitment

Stakeholders within the OSFB consortium are selected based on their specific expertise, professional roles, and capacity to contribute to the development, implementation, and evaluation of the program, ensuring alignment with the quadruple helix model (academia, industry, government, and society) [18]. Academic stakeholders were included based on their established research expertise in either periconceptional health, lifestyle, public health, or prior relevant collaborations. Governmental and public health stakeholders (including municipality, youth health care providers, and policymakers) were selected based on their formal roles in local health policy, implementation knowledge, and access to the target population. Industry partners were primarily selected based on their ability to contribute to the digital infrastructure, innovation, and scalability of the intervention. In addition to the initial stakeholders involved in the original grant application, the consortium remains open and adaptive, allowing for the inclusion of additional stakeholders, such as local entrepreneurs and community partners, throughout the program to further strengthen its impact and contextual relevance.

Stakeholders representing the societal domain, including the target population, are identified and reached through existing community networks, local initiatives, and the 4 field labs set up by WP2 in phases 1 and 2. Particular attention is given to including individuals who are embedded in the local community, including key figures, community and health care workers, representatives of local programs and initiatives, and (future) parents themselves. To ensure that the perspectives of the target population are well represented, we aim to involve a diverse group of participants from IJsselmonde reflecting variation in family composition and socioeconomic background. Potential participants will be informed about the study and recruited through key organizations in Rotterdam-South, social activities targeting vulnerable groups, social media, and other promotional initiatives. Members of the research team will attend these social activities to speak with potential participants and explain the study in person. Stakeholders interested in participating can visit the program website [36]. The potential users of the Family Buddy will be referred to the landing page [37] to obtain further information, register for the study, review the patient information folder, and provide informed consent. Alternatively, users can register and provide informed consent in person with a member of the research team or with an employee of one of the key partner organizations. All recruitment materials will be available in both Dutch and English.

### Data Collection, Sharing, and Analysis

#### Data Collection

Data collection within the OSFB program includes a combination of quantitative and qualitative methods. These include, for example, questionnaire-based assessments on lifestyle, sleep, and psychosocial factors among (future) parents, as well as qualitative data collection through interviews and focus groups conducted within the field labs. In addition, user data (eg, prototype usage and engagement metrics) are collected to evaluate user interactions and implementation of the intervention. All details on data collection for the separate studies are and will be described in more detail in the corresponding individual research papers [22-24,38].

All data collection and handling procedures are conducted in accordance with the Dutch Research Council data management policy. Within the OSFB consortium, a Collaboration Agreement and Data Management Plan have been established and are signed by all WPs, specifying procedures for data collection, storage, sharing, and governance (Supplementary info 2 in Multimedia Appendix 1).

#### Data Analysis

Standardized data analysis methods will be applied for the analysis of both qualitative and quantitative data. Data analysis will be conducted in alignment with the specific objectives and outcomes of each WP, using an integrated mixed methods approach.

For phases 1 and 2, qualitative data from semistructured interviews, field notes, and focus groups are transcribed verbatim, pseudonymized, and analyzed using ATLAS.ti. A thematic analysis approach following Braun and Clarke is applied [19], combining deductive coding and inductive coding. Coding is conducted iteratively, with multiple researchers independently coding an initial subset of transcripts to develop and refine a codebook; discrepancies will be discussed until consensus is reached. Identified themes are subsequently mapped onto behavioral frameworks, including the COM-B model and the Theoretical Domains Framework, to systematically categorize determinants of eHealth use.

For phase 2, qualitative data from interviews, focus groups, and cocreation sessions are analyzed using reflexive thematic analysis, complemented by template analysis to enable a structured yet flexible coding process guided by the research questions [39]. Concept mapping data are analyzed using multidimensional scaling and hierarchical cluster analysis to identify clusters of related ideas and priorities, while Q-methodology data are analyzed using by-person factor analysis to identify shared viewpoints among participants.

For phase 3, quantitative data from the pilot evaluation will be analyzed using both descriptive and inferential statistical methods. Descriptive statistics will summarize participant characteristics, usability scores, and engagement metrics (ie, login times, module usage, and days of usage of the Family Buddy). Changes in lifestyle-related outcomes, including nutritional behaviors and mental health indicators, will be assessed using 2-tailed *t* tests, chi-square tests, or nonparametric equivalents. Where applicable, multivariable linear and logistic regression models will be applied to evaluate associations between Family Buddy use and changes in outcomes, adjusting for relevant covariates, such as demographic and baseline characteristics. Subgroup analyses will explore heterogeneity of effects across demographic and baseline characteristics. App-based user data will be analyzed using descriptive statistics and longitudinal usage patterns, including regression models to explore time-response relationships between engagement and behavioral outcomes. Qualitative data from interviews and open-ended responses in the studies in phase 3 will again be analyzed using thematic analysis to understand user experiences, contextual factors influencing engagement, and perceived mechanisms of behavior change using the Family Buddy.

Across all 3 phases, triangulation will be applied by combining method triangulation (the integration of qualitative, quantitative, and app-based user data) and data source triangulation (comparison of perspectives from participants, health care professionals, local stakeholders, and key persons from IJsselmonde) [31-33]. In addition, regular interdisciplinary exchange between PhD researchers, senior researchers, consortium partners, and technical development teams will enable continuous triangulation during both data collection and analysis. Iterative collaboration between qualitative research teams and technical developers will ensure that emerging insights are continuously translated into the design and refinement of the Family Buddy. Together, these approaches will provide a comprehensive understanding of the Family Buddy’s impact and inform iterative optimization of the intervention.

## Results

### Study Population Characteristics

In phase 1, we selected a deprived area of Rotterdam (IJsselmonde) as our research, development, and implementation setting of the Family Buddy. This decision was informed by the demographic, socioeconomic, and health characteristics of the area, which indicate a high prevalence of potential vulnerability factors, aligning with our aim to engage (pre)pregnant couples and young families at increased risk for health inequities. Furthermore, we ensured that no other research projects were hosted in the neighborhood that could either overburden the population or compete with our initiative. Table 1 summarizes the characteristics of the population in IJsselmonde in comparison to the general population of the Netherlands.

**Table 1.** Demographics of IJsselmonde in comparison to (the general population of) the Netherlands, 2023 (sources: STATline, buurtatlas.nl).

Characteristic	IJsselmonde	The Netherlands
Population		
Total population (N)	61,180	17,590,672
Western migration background, n (%)	6895 (11)	1,909,408 (11)
Non-Western migration background, n (%)	27,375 (45)	2,529,492 (14)
Age (y), n (%)		
0‐15	10,840 (18)	2,712,461 (15)
15‐25	7105 (12)	2,158,241 (12)
25‐45	17,390 (28)	4,399,003 (25)
45‐65	15,100 (25)	4,795,514 (27)
≥65	10,745 (18)	3,525,453 (20)
Educational level, n (%)		
Low	16,880 (37)	3,565,510 (20)
Middle	19,420 (43)	5,520,240 (42)
High	8840 (20)	4,215,660 (32)
Households, n (%)		
Households with ≥1 child	10,335 (35)	2,739,000 (33)
Income		
Standardized income, mean	27,700	33,500
Difficulty providing for basic needs (%)	33.1	20.5
Lifestyle (%)		
Smoking	23	18
Alcohol use	41.6	56.9
Exercise <150 min/wk	61.4	53.2
Overweight (BMI >25 kg/m²)	59.8	51.7
Obesity (BMI >30 kg/m²)	16.5	22.4
Mental health (%)		
Stress	30	20.7
High risk for anxiety and/or depression	16.1	10.2
Loneliness	62	49.2
Severe loneliness	21.6	14.4
Very low resilience	24.8	17.3
Obstetric characteristics (%)		
First antenatal visit after gestational age 10 wk	44.1	27.3
Preterm birth and/or small for gestational age (Big 2)	18.8	15.2

We defined the target population as women or couples with an active child wish, pregnant, or parents of at least one child below the age of 4 years, residing in IJsselmonde.

To assess the characteristics of our study population, a questionnaire was developed to assess (un)favorable lifestyle behaviors, mental illnesses, stressors, and other factors. This questionnaire is based on the Rotterdam Reproduction Risk Reduction questionnaire, a validated questionnaire used in health care practices in the Netherlands to assess the presence of potential vulnerability factors and unfavorable (lifestyle) behaviors [40,41]. Furthermore, we selected nutrition as a lifestyle target and stress reduction as interrelated targets to be improved by using the Family Buddy.

### Consortium Building and Stakeholders’ Involvement

The OSFB program has focused on building the quadruple-helix consortium within the neighborhood in phase 1, creating visibility and gaining trust, and inviting (pre)pregnant couples and young families to participate in interviews and cocreation sessions. A large consortium of more than 40 partners has been established and is sustained through regular meetings and collaborative sessions. By June 2025, more than 30 (pre)pregnant couples and young families were interviewed, and a user board was installed. Inclusivity of the program was increased by recruitment of families and parents within the neighborhood and offering the option of having the interview at the place of choice of the participants. Another 20 additional stakeholders, including health care professionals and local key persons, engaged in cocreation. Furthermore, 300 women contemplating parenthood have finished the digital *Pregnant Faster* study.

### Research Insights and Implications for the Family Buddy

Phases 1 and 2 of the program have generated valuable insights and research outputs across the WPs, laying the groundwork for the development and implementation of the Family Buddy. Table 2 summarizes the research aims, activities, and outcomes. While the initial grant proposal and first methodology of the OSFB program outlined the development of a primarily digital platform embedded within a social environment, the program activities and insights during phases 1 and 2 revealed that a stand-alone digital platform would not sufficiently address the social, relational, and contextual barriers experienced by the target population.

**Table 2.** Research methods, outcomes, and current status (December 2025).

Research aim	Research method	Study population and key outcomes	Status
Phase 1			
Explore existing eHealth tools	Scoping review	Vulnerable pregnant women Identification of key functionalities of eHealth tools for families in deprived circumstances	Published [35]
Explore implementation strategies	Systematic review (PRISMA^a^)	Deprived neighborhoods or circumstances, Dutch health care setting Identification of best practices for implementation strategies	Study in preparation
Identify the preferences of the target population	Semistructured interviews (COM-B^b^ model and Theoretical Domains Framework)^c^	Families in deprived circumstances Insights into facilitators and barriers to eHealth use among the target population	Manuscript submitted
Develop field labs methodology	Literature review	Methodological guidelines for field lab development	Manuscript in preparation
Develop an ethical framework	Narrative literature review and interviews^c^	An ethical framework to guide the development and implementation of the Family Buddy	Published [38]
Phase 2			
Implementation of field labs	Action research and participatory observation	Citizens and organizations active in IJsselmonde Report on first insights gathered during the field labs	Manuscript in preparation
Context mapping	Semistructured interviews using an interview guide, sensitizing exercises, and probes (cards)^d^	Families in deprived circumstances Insights into values, value tensions, and challenges regarding lifestyle changes	Published [39]
Integration into health care practices	Focus groups and interviews	Health care professionals Insights into facilitators and barriers for integration of the Buddy within the existing health care system Input for the content and design of the Buddy	Planned (2026, Q4^e^)
Concept development	Cocreation sessions^d^	WPs^f^ and project partners Transform insights gained through all WPs into a Family Buddy Families in deprived circumstances Ensure alignment of the prototype with the needs of the target population Enhance social cohesion and sense of community ownership of the prototype	Ongoing until Q2 2026^e^
Develop and evaluate usability of Pregnant Faster	Observational study^g^	Women with an active wish to conceive Insights into the usability of the nudging-based preconception application Pregnant Faster	Manuscript in preparation
Evaluate the effectiveness of Pregnant Faster	Observational study^g^	Women with an active wish to conceive Insights into the effectiveness of the nudging-based preconception application Pregnant Faster	Manuscript in preparation
Understand users’ mental models of generative conversational search and assess the effect of interface transparency	Mixed methods study^h^	16 experienced users of generative conversational search (early adopters) Users hold abstract, incomplete mental models (“black-box” perception) Transparency improves interpretability but can reduce satisfaction and increase cognitive load Trust is volatile; users rely on hybrid search workflows for verification Misaligned mental models affect appropriate trust and system use	Manuscript in preparation
Identify and formalize evaluation metrics for LLM^i^-based conversational search	Qualitative study^h^	11 health care professionals (the Netherlands) Developed a 2-part evaluation framework (Knowledge and Interaction metrics) Identified key human-centered metrics, including context-seeking, empathy, and trustworthiness Demonstrated limitations of automated evaluation metrics in health care Highlighted the need for expert-driven, domain-specific evaluation approaches	Manuscript in preparation
Investigate how conversational agent persona influences source-clicking, trust, and reliance	Between-subjects quasi-experimental study^h^	57 participants with varying health literacy Persona significantly affects verification behavior Health literacy moderates verification behavior Persona has limited direct effects on trust and perceived accuracy but indirectly influences appropriate reliance Overall low source-clicking rates highlight a key design challenge	Published [42,43]
Phase 3			
Evaluate real-world engagement, usability, and short-term effectiveness	Mixed-method pilot study^j^	Quantitative component: 50 participants from IJsselmonde Lifestyle behavior changes Changes in mental health Actual Family Buddy usage User interaction patterns, frequency of active days, and login behavior Qualitative component: 15‐24 participants In-depth insights into how, why, and under which circumstances the Family Buddy is used and integrated into daily life	Quantitative: Planned (2026, Q3^e^) and Qualitative: Planned (2026, Q3^e^)
Evaluate improved Family Buddy and implementation	Observational cohort study with an embedded RCT^j^^,^^k^	200 families and future parents from IJsselmonde Insights into implementation processes Insights into behavioral change mechanisms	Planned (2027‐2028)

a PRISMA: Preferred Reporting Items for Systematic Reviews and Meta-Analyses.

b COM-B: capability, opportunity, motivation, behavior.

c Medical ethical approval granted by Erasmus MC University Medical Center Medical Ethical Committee, identification number MEC-2024‐0268.

d Ethical approval granted by TU Delft Human Research Ethics Committee, identification number HREC-3420.

e Q2=second quarter of year (April to June), Q3=third quarter of year (July to September), Q4=fourth quarter of year (October to December).

f WP: work package.

g Medical ethical approval granted by Erasmus MC University Medical Center Medical Ethical Committee, identification number MEC 2020‐0974.

h Ethical approval granted by TU Delft Human Research Ethics Committee, identification number HREC-3459.

i LLM: large language model.

j Application for Medical ethical approval at the Erasmus MC University Medical Center Medical Ethical Committee (METC) will be done prior to start study.

k RCT: randomized controlled trial.

The cocreation process was informed by 4 complementary substudies that explored families’ lived experiences, needs, and perspectives from different angles, thereby increasing context specificity and strengthening the conditions for future implementation. The first qualitative substudy explored parental value tensions related to nutrition decisions for young children among 20 parents living in disadvantaged neighborhoods. This study demonstrated that parents frequently experienced tensions between the dietary health of their child and competing values, such as child enjoyment, parental convenience, and parental well-being, particularly in situations characterized by stress, financial strain, limited social support, or challenging child behavior. Parents described social and material support, including informal household help and access to healthy and convenient foods, as important protective factors that reduced stress and facilitated healthier nutrition choices. These findings highlighted the importance of designing interventions that are sensitive to parents’ daily realities and underlying value conflicts [38]. The second qualitative substudy, conducted by the Department of Neonatal and Pediatric Intensive Care of the Erasmus MC - Sophia's Children Hospital, explored barriers and facilitators for engagement with eHealth lifestyle interventions among 15 parents (13 women and 2 men). This study demonstrated that engagement with digital lifestyle support is shaped by factors such as digital accessibility, trust, motivation, and alignment with families’ daily realities. Ethical approval was obtained from the Erasmus MC Ethics Committee (MEC-2024‐0268). The third substudy, conducted by Delft University of Technology, explored parents’ preferred forms and delivery of social support related to parental well-being and healthy child nutrition among 19 parents (17 women and 2 men). Findings highlighted the importance of tailored informational support combined with locally embedded emotional and practical peer support. The fourth substudy, conducted by the Section Medical Ethics, Philosophy and History of Medicine of Erasmus MC, evaluated the acceptability of different forms of influence within digital lifestyle interventions among 16 parents (14 women and 2 men). Parents considered digital influence acceptable when it was transparent, motivating, effective, and preserved freedom of choice and autonomy. The latter 3 studies are submitted for publication. Together, these substudies addressed complementary social, behavioral, practical, and ethical dimensions that are essential for the cocreation, acceptability, and future implementation of a meaningful socially embedded digital lifestyle intervention for families living in vulnerable circumstances.

Insights from the literature reviews, the qualitative substudies, and the cocreation sessions consistently highlighted the need for stronger interpersonal support within the neighborhood, involving local key actors and existing networks within the community. In response and as a result of iterative development cycles, the program’s focus shifted from a predominantly digital Family Buddy concept toward a more integrated socially embedded-digital intervention that combines an individual digital Family Buddy lifestyle coaching platform with a structured and supporting social component consisting of group meetings and workshops in the local environment.

## Discussion

### Anticipated Findings

This paper describes the methodology and summarizes the first research insights from the OSFB program, with the primary aims of improving nutrition and sleep in young (future) families living in deprived circumstances. The first 3 research years, spanning phase 1 and (part of) phase 2, provided key insights into consortium building, engagement of the target population and their needs, the establishment of community-based field labs, and the development of an ethical framework for OSFB.

A key finding from the first 3 research years is the need for strong societal embedding of the OSFB program by implementing in already existing community initiatives. One of the field labs organized by WP2 is meal prep programs, during which inhabitants of IJsselmonde cook together while discussing topics relevant for a healthy lifestyle. These programs are guided by professional chefs. Other examples include parent-child groups and involving key community figures through train-the-trainer approaches. This led to the development of a socially embedded digital Family Buddy. Rather than targeting systemic change, the OSFB program emphasizes the role of small, supportive communities in fostering sustainable behavior change, with the social-digital Family Buddy supporting both individuals and community.

### Interpretation

Literature reviews from phase 1, as well as interview studies in phase 2, conclude that current digital tools to change, adopt, and sustain healthy lifestyle behaviors and support coping with mental stress show limited effectiveness in reaching and benefiting underprivileged (pre)pregnant couples and young families [22]. This may not only reflect gaps in user-centered development but could also relate to the broader reality that many couples and families face systemic stressors, which might reduce their cognitive and emotional capacity to meaningfully engage with such tools. In addition, some individuals, couples, and families may experience limited trust in formal institutions, particularly when (digital) tools are perceived as extensions of those institutions. Research suggests that a sense of not being recognized or mentalized can hinder the development of epistemic trust, which is critical for engagement and uptake of supportive interventions [44]. Many tools on the market have been developed without the direct involvement of the target population as well as other involved stakeholders, leading to low user engagement and usability issues among these groups [14]. Studies indicate that tools not developed specifically for or with underprivileged populations often face barriers in implementation, as they may not adequately consider the unique needs, values, challenges, and environment faced by these users [15]. Consequently, such tools often fail to integrate into the users’ daily routines and do not achieve the desired health outcomes [45]. Ultimately, these tools exacerbate health inequities, as underprivileged populations are unable to benefit from them while their wealthier counterparts may gain increasing health advantages.

The cocreation process and field lab method are central to the development and societal embedding of the Family Buddy and its broader implementation strategy. By involving women, couples, families, health care providers, and local stakeholders throughout the design and evaluation stages, the Family Buddy becomes more aligned with the needs of families living in deprived neighborhoods [16,46]. However, long-term uptake and ownership cannot rely on field labs alone. Sustainable success requires active collaboration, trust building, and shared responsibility with community partners. Early signs of ownership are emerging—for example, local organizations are co-hosting activities and expressing interest in continued use—but formalizing these partnerships and exploring long-term governance models remain priorities. Further research is needed to understand how to achieve lasting community ownership beyond the project phase.

### Strengths and Limitations

The OSFB program has several strengths and limitations. The key strength is its large, transdisciplinary quadruple-helix consortium and its comprehensive cocreation design process embedded within existing communities in a deprived neighborhood. By engaging the community and the target population involved through cocreation and field labs, the program aims to explore and address the needs of underprivileged populations, resulting in a comprehensive and well-accessible tool. Another strength is the exploration of the integration of a conversational agent for real-time tailoring of advice based on the user’s needs and input, a feature likely to enhance engagement and effectiveness [45]. An additional strength is the ongoing critical exchange of knowledge and experience within and between the WPs, which has led to new insights that contribute to the development of a complementary social intervention within OSFB to address collective and environmental factors alongside individual support.

This program faces several limitations as well. First, the inherent complexity of coordinating a large transdisciplinary consortium, where progress depends on a shared vision, mutual trust, ownership, and alignment among diverse stakeholders, poses a significant challenge. Moreover, it is difficult to integrate all insights into clearly defined cocreative designs and field labs; to address this, we will organize sprint sessions within the consortium to better delineate the process and enforce alignment. In addition, the resource- and time-intensive nature of our extensive cocreation and iterative field lab methodology may limit the generalizability of our findings to other contexts. The reliance on digital access and literacy could also restrict the reach of the Family Buddy among subgroups lacking internet connectivity or smartphone access [15]. Another limitation lies in the complex implementation context for (pre)pregnant couples and young families, who are supported not only by health care professionals but also by a wide range of stakeholders within the social domain. These systems often operate in parallel with limited coordination, making it challenging to ensure continuity, alignment, and shared responsibility across sectors, a known barrier in integrated and person-centered care implementation [47]. Finally, the actual development and maintenance of the platform when the OSFB research program concludes demand substantial infrastructural investments and ongoing costs, especially which may constrain scalability if not managed effectively. To ensure sustainable evaluation and improvement, the digital functionalities included and developed within the Family Buddy have to provide secure access to usage data and include clear agreements on data ownership and ownership of OSFB, safeguarding transparency and long-term usability beyond the research program.

### Conclusions

The OSFB program aims to develop a socially embedded Family Buddy to support coping with mental stress and improve nutrition within a supportive living environment for (future) parents before and during pregnancy and families with at least one child under the age of 4 years. The program combines cocreation with existing interventions tailored to the needs of families living in IJsselmonde, a deprived neighborhood in Rotterdam. By involving the target population and community stakeholders at every stage, the program aims to ensure that the Family Buddy is relevant, accessible, and capable of supporting sustainable lifestyle changes. This tailored, user-centered, and life course care approach intends to foster long-term health improvements and social cohesion within communities, positioning the program as a scalable model for reducing health disparities and enhancing quality of life in deprived neighborhoods in the coming years.

## Supplementary material

10.2196/88913 Multimedia Appendix 1 Interview guide.

10.2196/88913 Peer Review Report 1

## Abbreviations

COM-B: capability, opportunity, motivation, behavior
LLM: large language model
NWO: Dutch Research Council
OSFB: Our Smart Family Buddy
WP: work package
