# Supplementary material for: Socially Embedded Digital Coaching Platform to Promote Healthy Lifestyle and Mental Health in Underprivileged (Pre)Pregnant Couples and Young Families (Our Smart Family Buddy Program): Protocol for a Mixed Methods Study
Source: JMIR Res Protoc. 2026 Oct 15;15:e88913. doi: 10.2196/88913 (PMC13671683; doi:10.2196/88913)
Supplement: Supplementary file 1 [file resprot-v15-e88913-s002.pdf]

Aanvraag

**Titel**	“Our Smart Family Buddy” Co-creative Interactive Digital Buddy platform operating by App and robot (Avatar) for young families in deprived neighbourhoods that fosters health literacy, healthy lifestyle, and healthy environments.
**Aanvrager**	prof. dr. R.P.M. Steegers-Theunissen
**Dossiernummer**	KICH1.GZ01.20.016

1 - Problem posed and problem analysis, contribution to solution

a

What is your opinion on the scientific and societal relevance of the research problem and the innovation question(s) posed?

Commentaar referent 1

The societal relevance of this proposal is quite strong. Currently globally the issues in deprived neighbourhoods needs to be explored more and this proposal would do exactly that. From a scientific standpoint focusing on my area which is Computer Science/Engineering, it is hard for me to gauge the scientific relevance of the innovation as the technical methodology that would be common in my area are absent from the proposal. I would lean towards the experts in health research to comment on the scientific relevance.

I will add that in a local context focusing on a problem that exists in the Netherlands, this proposal could have significant impact.

Commentaar referent 2

The research problem is highly relevant as far as the scientific and societal context is concerned. Use of data analytics methods like Artificial Intelligence (AI) and Machine Learning (ML) techniques for healthcare application is one of the most sought-after field of research and I professionally believe it may have promising outcome provided the data-set is of high quantity as well as quality. However, the project proposal does not provide much of an insight to the technological aspects of the project rather a well-structured high-level strategy and planning document. The overall innovative strategy proposed in the full report is adequately elaborative however, as an engineer I am not in a position to comment much about the engineering design (Big-data, Data Analytics: AI / ML) aspect of this proposal as not much engineering design aspects are presented in the proposal. Last but not the least, commenting from technology aspect the key to the successful implementation of this project predominantly depends upon the high-quality and large data-set as most of the AI and ML based system works on the data-set. The exploration of AI and ML algorithms may not be that challenging as data acquisition from real-world scenario may pose several societal, ethical and legal challenges.

Commentaar referent 3

I believe that the scientific and societal relevance of the research problem and innovation question is very high.

Weerwoord

@ref1: Next to our scientific impact on the lifestyle and living environment research disciplines, we also foresee contributing to advancing the Computer Science field, specifically in the area of Human-Computer Interaction. Through training of chatbots and deploying them with young families over long periods, we will elicit design guidelines for conversational interfaces, test new conversational interaction approaches, and evaluate chatbot personality of behavioral change. In particular, scientific relevance relates to the use of Computer Science methodology in highly sensitive data that requires Privacy Preserving Techniques (WP4).
@ref2: In WP4, we will apply statistical modelling and state-of-the-art machine learning methods such as gradient boosting and random forest for risk prediction. A large national cohort is available comprising health, income, therapy-use, education, environmental and maternity care data for children and their parents hosted by Statistics Netherlands (CBS) to train our prognostic models and determine the most impactful risk factors leading to negative health outcomes. In addition, latent class analysis will be used to find meaningful profiles of groups with similar characteristics that can be the focus of targeted interventions. We will collect data relevant for the defined Key Performance Indicators for healthy lifestyles using various probes from our partners. Collected data includes, but is not limited to, physical activity levels (e.g., stepcounts), lifestyle behaviours (e.g., www.smartereatingwithyourchild.co.uk), visited locations (e.g., home/work), app-usage, etc. With wearable devices, we will collect additional physiological data such as heart rate, electrodermal activity and sleep quality. This information will be used to produce a lifestyle-index that drives the intervention and interaction with the ID-Buddy. The data collected is stored in a scalable data management system. The high-quality is assured by storing the data structured and in a way that the data applies to required data format standards and is immediately available for research. The value of

the data for AI techniques arises over time as the size grows through the longitudinal collection of data. We believe that through our learning communities/field labs societal challenges in working with young vulnerable families can be overcome. Furthermore, we have a dedicated WP6 on Ethics run by ethical experts.

b

To what degree are the research questions and objectives of the research articulated in collaboration with relevant partners and stakeholders?

Commentaar referent 1

The RQs were given through a series of Objectives (1-7) leading to WPs (1-6).

The RQs were well articulated with strong collaboration with other stakeholders. The proposal was quite diverse and collaborative with a lot of collaborators over the 6 year period of the grant.

Commentaar referent 2

Research questions and objectives are well articulated in collaboration with relevant partners and stakeholders. I am highly satisfied in this context.

Commentaar referent 3

The research questions are well formulated and it seems, but I can not really assess that) that they are formulated in collaboration with relevant partners and stakeholders. I only miss scientific expertise in the field of health inequality and community interventions. Furthermore, to me it seems that the research question is very much technology driven and the basis for using this technology not well explained nor supported by the literature. This does not mean it is important, but there are merely assumptions.

Weerwoord

@ref3: Coordinator Steegers-Theunissen and Evers (WP1-lead) are scientific experts in eHealth and community implementation of lifestyle interventions. From 2006 onwards reducing health inequalities in the city of Rotterdam (multi-cultural city characterised by socio-economic and health inequalities) is the mission of multiple (societal) experts working together with young vulnerable families on a daily basis (section2.2.3) on projects such as HealthyPregnancyforAll, SolidStart, HealthyStart, ResilientDelta. Moreover, in Rotterdam there are already positive experiences with conversational chatbots for people facing problems in regular communication about health (e.g. mentally challenged, people with ASS). The past 2 years our experiences with the application of chatbots in mental health e-health modules from the New Health Group (an ORTEC business unit) shows a better response from clients/patients (users) to chatbot messages than expected. These experiences will be researched and the knowledge gained will be used in the development of the chatbots for 'Our Smart Family Buddy'.

c

To what degree will the proposed research contribute to solving the innovation question posed and the societal challenge(s) as described in the introduction above and further detailed in the call for proposals?

Commentaar referent 1

The innovation questions are somewhat solved with the proposal.

One lacking component that was made clear in Section 2.1 of the CFP was Artificial Intelligence. There could be room in the grant for such innovation but details is absent from the innovation as described. This addition would also add significant novelty from a Computer Science/Engineering aspect. Such methodology for how AI would be used exactly is absent. Details of such innovation should have been added.

Commentaar referent 2

The proposed research contribution very well presented with an elaborative strategy and implementation plan to address innovation question posed with societal challenges.

Commentaar referent 3

The proposed research wants to use an ID buddy to change the lifestyle of young families by increasing health literacy and also reduce environmental risk (see societal impact). It is not clear from the proposal on how they want to achieve the latter goal, other than involving them in the proces.

Furthermore, the search is on a technology-driven solution, but the problem of health inequality is very complex and only described in general terms. And the question remains if this technology can provide a solution (as the authors claim) to this problem. An important assumption is that young families in disadvantaged positions want to change their lifestyle and would be helped by an ID Buddy. The research to support that is completely lacking. Until now many interventions have so far failed to reduce health inequalities. We know why these interventions fail and a technological solution is not necessarily the answer. It seems that there is insufficient connection to the existing literature.

Weerwoord

@ref1: WP4 aims at producing “with-AI” contributions, where AI is applied to facilitate societal change and societal innovation. Our research on Human-Computer Interaction will deliver novelty to the field of Computer Sciences (see reply to

comment 1b ref 3).

@ref3: Social ecological guidelines will be used as a way to realize individual and environmental impact. For example, we will use personal health and CBS-data to identify individual, community and institutional drivers of health, and then develop and evaluate socioecological interventions within a focal living lab – learning community. In short, the guidelines based on ecological principles are (Stokols Am.J.Hlth.Prom.1996):
1.Examine links between multiple facets of well-being and diverse conditions of the sociophysical environment.
2.Consider joint influence of intrapersonal and environmental conditions on individual and community well-being.
3.Develop health promotion programs that enhance fit between people and their surroundings.
4.Focus health promotion interventions on high-impact behavioral and organizational "leverage points".
5.Design health promotion programs that address interdependencies between the physical and social environment and encompass multiple settings and life domains.
6.Integrate multidisciplinary perspectives in the design of health promotion programs and use multiple methods to gauge scientific and social validity of interventions.

Although we have positive experiences with the uptake, compliance and usability of our eHealth lifestyle coachingprograms in individuals with low-SES (PMID: 30973345), ref3 is correct in stating that most eHealth insufficiently realises uptake by these citizens. The problem is that eHealth is often designed in a one-size-fits-all aim and does not align to specific interest, needs and context of the users with a low-SES. Therefore, most solutions created health disparities. In our related national project Benefit4All, we are developing eHealth guidelines for low-SES that turn this problem into a solution. These guidelines are validated and based on positive experiences of eHealth developers/care providers and on healthcare expectation and health attitudes of people with low-SES. Secondly, we have promising evidence on the effect of interactive storytelling (https://www.tudelft.nl/io/onderzoek/health/healthy-storytelling) to engage people with low-SES into eHealth interactions, which will be applied as a driving element in the ID-Buddy.

2 - Expected impact

a

To what degree do you expect that the proposed research will have societal and/or economic impact?

Commentaar referent 1
I feel if fully executed,looking at the RQs given through a series of Objectives (1-7) leading to WPs (1-6), the proposed research would have a well established and repeatable societal and economic impact.

The WPs are well laid out with a timeline and personnel plan fitting for the research to be conducted.

Commentaar referent 2
I professionally believe the proposed research proposal has a very good potential towards an incremental societal and economic impact at large. However, the success of this project largely depends upon quality and data-set acquired from user community. While evaluating the proposal the NWO should take section 2.8 (Risk management and contingency planning aspect) into account.

Commentaar referent 3
see previous remarks. There are quiet a few assumptions (see 2,1,4,), that are not supported by literature and if they do not hold, there will be no impact.

Weerwoord
We thank ref1 for the positive feedback. We agree with ref2 that data quality is critical for success. Our access to well-defined datasets and experience in building high-quality datasets will mitigate the risk of poor data quality. To ref3, we reply that it is inherent to making assumptions that these may not hold. Our assumptions for the expected societal impact have been defined in a joint effort and are based on extensive relevant experiences from our consortium (see also 1.b.). Through our impact pathway, we will continuously monitor if our assumptions are still valid and adapt if needed.

b

What is your opinion on how the proposal involves relevant stakeholders and end users in its route to impact?

Commentaar referent 1
The proposal does a very good job involving relevant stakeholders at the various stages of the proposed research. One concern I had was how exaclty (more details) how the personnel meant to be hired in the proposed research would be properly guided. Methodological details were absent in these aspects of the proposed research.

For example:

What will the 5 PhD student do over his 6 years, who exactly will guide him over that period? What are his/her expected outcomes over the 4 years. Are there any fail safes in place if things do not go as planned?

Commentaar referent 2
The proposal has a well-planned route to impact with well formulated road-map involving all stakeholders and end users. The outcomes as well as outputs of the proposal has been well articulated in section 2.3 with great details.

Commentaar referent 3
This is well done. A lot of important parties are aligned and they also contribute financially and content wise. So if the ID buddy works the chance for wider implementation is very positive.

Weerwoord
@Ref1: The local institutional supervisors are primarily responsible for the recruited PhD-students and postdocs. The WP-leads will provide further guidance by arranging for structured meetings. We will also create room for ad-hoc meetings if needed. We thank refs 2 and 3 for their positive feedback.

3 - Quality consortium

a

What is your opinion on the track record of the consortium partners, both at a scientific level but also with respect to knowledge utilisation and application of research results?

Commentaar referent 1
The team is large, with one Principal Investigator, and 12 co-applicants for various portions of the grant.

Prof. R.P.M. (Régine) Steegers is a strong academic with an excellent track record, the applicant is well suited to lead this proposed research.

Focussing on WP4, which would be my main area of the proposed research, the two main leaders/applicants, namely Dr. E. Niforatos and Prof. dr. ir. W. Kraaij, have adequate background in the area to lead this part of the proposal. It would be have been beneficial to add a co-applicant with a stronger background in Health-AI, however the reviewer understands there may be limitations to who can be added to such grants.

Commentaar referent 2
I believe the team is quite good. To be professionally frank, I don’t know them directly but I found the consortium partners are quite competent as per my brief research over World Wide Web (Internet) sources.

Commentaar referent 3
The track record of some, but not all of the scientist is impressive. It is difficult to say what their expertise is with respect to knowledge utilisation. But they do not have a specific scholar, as far as I can assess, who is particularly skilled at translating knowledge to practice.

I am particularly impressed with the many partners and it is certainly very interdisciplinary.

Weerwoord
@ref1: We acknowledge the feedback and have invited Dr.ir. Willem-Paul Brinkman (TU Delft, Computer Science) to join as an advisor. He has a strong background in health-AI with relevant expertise on human-computer interaction, human-centred AI, behaviour change support systems, and the use of artificial social agents.
@ref3: Several partners have a track record in translating knowledge into practice. Coordinator Steegers-Theunissen has developed and released several eHealth lifestyle coaching applications. Prof.Joosten has translated scientific results in (inter)national clinical guidelines and implementation of guidelines locally and nationally. Prof.Evers is expert in changing behaviours and developing implementation strategies in the society. RSM will set-up a Center for Managing and Marketing Innovations in which academics and entrepreneurs can meet to share knowledge and experiences for the purpose of implementing innovations. The TU-Delft Dept of Human-Centered Design (Visch) and Dept of Design, Organisation and Strategy (Bosch-deVos) have extensive experience in bringing (psychological, health, design) theory into practice (co-creation, iterative prototyping) in a multi-stakeholder context. Improve as innovative and experienced event organization will contribute to the implementation (Strategy).

b

Does the research team contain the relevant and necessary (inter)disciplinary expertise for the proposed project?

Commentaar referent 1
This proposed research is strongly inter-disciplinary, mixing Computer Science, Health, Medicine, Software, Nutrition, and Industrial components.

Overall, looking at the Table in 2.2.3, the applicant and co-applicants have gathered an over strong interdisciplinary team to conduct the proposed research.

Commentaar referent 2

I believe yes. (Disclaimer: I have no direct knowledge about their professional competency, however from their track record and prior work available over internet, I believe so.)

Commentaar referent 3

This is a very interdisciplinary team. I like very much the expertise and hence the WP on ethical and legal issues. However, the scientists and WP leads of the consortium are mostly people with medical and technical backgrounds. There seems to be a lack of knowledge and expertise among the researchers (not so much among the partners) in the field of reducing health inequalities and pedagogy. I infer this also from the fact that so little knowledge that is already present is described in the proposal.

Weerwoord

@ref3: Our project focuses on young families in deprived neighbourhoods, given the high potential for long-term lifestyle change in this group. We have assigned WP-leads with expertise among others behaviour change, implementation, ehealth, data science, and design, including earlier projects on health inequalities around pregnancy, or are embedded in research groups with health inequality or children experience (see also 1.b). Combined with co-creation and co-design with the stakeholders and end-users in fieldlabs, this will make sure that the implementation fits the target group. Together with the overall expertise of our consortium, we are convinced that the appropriate knowledge is in place. Notably, the involved expertise of the TU-Delft Industrial Design Faculty should be categorized as a design partner by having a large expertise and explicit research aims in multistakeholder design, human centered design, and design in the medical context. TU-Delft partners have developed several experience-based methods to reach and involve difficult to reach groups in design processes and outcomes, aiming at health inequality reduction.

c

Are all relevant academic and non-academic partners present in the consortium, and is their role in and contribution to the project clear and convincing?

Commentaar referent 1

Yes, the proposed research has laid on properly all academic and non-academic parts and roles and contributions well.

Backup (contingency) plans are absent from the proposed research.

Commentaar referent 2

Yes, academic and non-academic partners in the consortium and their role in the project is quite clear and convincing.

Commentaar referent 3

Again I miss some expertise, but the role of the partners has been made clear. However I have serious concerns about the alignment of the work packages (see also later)

Weerwoord

@ref1: Our risk mitigation measures are described in section2.8. Given the experience of our consortium, and embedding of the project in several ongoing initiatives (e.g.Diaper: Codeboek DIAPER | RIVM), we have several opportunities and directions for the project to succeed. For example, the whole endeavor will be largely supported by the Design-at-Scale(D@S)-lab in place at TU-Delft. The D@S-lab investigates novel forms of human-AI collaboration for tackling complex societal problems, such as lifestyle choices of underprivileged families.
@ref3: see response on comment 3a, and 4a.

d

What is your opinion on the manner in which the required co-funding by public and/or private parties has taken shape in this consortium?

Commentaar referent 1

In 2.5.3 if the proposal, we see a good mix of in-kind donations, and public and private contributors. The applicants have gathered strong buy-in for this proposed research and that is commendable.

Commentaar referent 2

The proposal of co-funding agencies is mentioned in section 2.5.3 and its quite satisfactory. However, some private co-funder’s commitment letters are unavailable.

Commentaar referent 3

This looks good, as far as I can assess this.

Weerwoord

@ref2: We have sufficient co-funding to execute the project and to meet the grant guidelines. However, as mentioned in &2.5.3, more private and public partners expressed their interest in our project proposal, but needed more time to arrange the commitment letters.

e

Does the proposed collaboration in the consortium provide added value? Will the composition of the consortium result in synergy and complementarity?

Commentaar referent 1

The proposed consortium of collaboration has strong chain-wide collaboration. The proposed research makes good use of Human Capital and plans to train a collection of PhD and Post-Doc students over the 6 years. There is strong diversity on the private, public and civil society partners involved.

The proposal does a very good job laying out who and when (timelines) will work on this research and what the expected outcomes will be.

Commentaar referent 2

Yes, I believe so. However, I must admit I don't have any prior first hand (direct) knowledge regarding the member organizations part of the consortium.

Commentaar referent 3

Yes I believe so. They have managed to get all necessary partners on board.

Weerwoord

We thank the refs for their positive comments.

4 - Quality research

a

What is your opinion of the scientific and methodological quality of the proposal?

Commentaar referent 1

From my expertise, I can only comment on WP4, where I feel the details of implementation and methodology to be used for this WP is lacking in the proposal. The proposed work is good, I would have like to have seen more details on how the applicants planned on conducting this part of the research.

I would have also like to have seen contingency plans if the proposed research did not go as planned

Commentaar referent 2

I believe the scientific and methodological quality of the proposal is very good. The use of data analytics (AI) for healthcare application has proven to be quite promising with current research scope. However, it would have been better if the proposal to have an initial technological / engineering sketch on the types of data, healthcare parameters, sensors, health trackers, devices, algorithms etc. Without an engineering system overall initial design blueprint, it would not be possible from my side to comment much on the practical engineering aspect form system implementation point of view. But the overall idea has good potential.

Commentaar referent 3

The quality of the work packages look good, although I do not have the expertise to assess the quality of WP3. But I do have some questions and remarks:

Questions:
Overall, I do not really understand what they are going to do with machine learning and how they are going to make predictions based on neighborhood data or how they want to incorporate existing apps. That part could be better described.
Furthermore, I do not understand how the ambition to influence both the individual/family and the environment is reflected in the approach of the work packages (2.1.3 societal impact bullit 2). I do see involvement of the environment but not how they are also helped/influenced by the ID Buddy.

Remarks:
I like the use of learning communities, although I would like to know more about that concept.

Structure of the work packages: The work packages are described in a relative separate way. There are references to each other but it looks like that everyone has described their own package and there is not a proper alignment, other than a visualization. I think that is a shortcoming and also a risk in implementation.

Furthermore the work packages are conducted by scholars of different disciplines, which in itself is good, but how do they ensure a good connection between the WPs when we know that there are big differences in research culture and knowledge. It might have been better to have people with different disciplines working in teams. Furthermore, I find it very unclear how work package 4 relates to 3 and also actually 2. Because young families are involved everywhere. And I also do

not understand the mentioning of KPIs (WP 4). is that meant to be able to evaluate in the end? But that doesn't come back in WP6? Because they only work with WP1, and WP3 and WP5. Please explain.

Weerwoord

We have answered the concerns of ref1 on the methodologies in our responses above. Since all data is available, risks for the data science are very low.
@ref2: Although we will use already available solutions/products, it will take some effort to design a blueprint for both apps that will be used for data collection and for data itself. Therefore, this is part of the project plan. The information will be collected by the probes which we will deploy and provided largely by our partners. Mobile apps will be deployed on our participants' devices for collecting automatically behavioral data such as physical activity, visited locations. Wearable devices (e.g., EmpaticaE4) will collect information about the physiological-state. This information will be used to elicit a "lifestyle-index" based on which our ID-Buddy will provide customized consultation. Machine learning on the national CBS cohort will be used to find salient risk and protective factors leading to relevant health outcomes. Latent Class analysis will help to find meaningful clusters in certain neighbourhoods. In this way, our solution can be tailored to specific groups by focusing on diminishing the (modifiable) risk and augmenting protective factors to increase the effectiveness of blended care.
@ref3: We appreciate the concern about the management of a consortium with a high level of multi-disciplinarity, which we believe is also a strength. To promote intra- and inter-WP interactions, we will organise regular WP and consortium meetings. The coordinator together with a dedicated project manager will closely monitor if WPs interact and if there is mutual understanding. Through monthly meetings of the Steering Team composed of all WP-leads, the inter-WP alignment and engagement is governed and warranted (see section 2.4.2). It is important to note that this project builds on existing collaborations between participating medical, social and technological scientists who are part of Medical Delta and Convergence research communities.

b

To what degree is the proposed research urgent and innovative?

Commentaar referent 1

The proposed research overall is quite innovative and timely. I have also seen globally issued in under-privileged areas lacking the ability to be technologically advanced through lack of access alone. The Interactive Digital Buddy platform would help alleviate some of the gap I can say without ua doubt.

Young families in deprived neighbourhoods are often deprived of health literacy, healthy lifestyle, and healthy environments. The proposed research would solve that potentially;.

Commentaar referent 2

From the perspective of technology, use of Artificial Intelligence (AI) in Healthcare application is quite innovative and urgent.

Commentaar referent 3

yes I believe it is urgent and innovative

Weerwoord

We thank the refs for their positive comments.

c

What is your opinion on the interdisciplinary aspects (if any) of the proposal?

Commentaar referent 1

Interdisciplinary aspects are at the crux of the proposed research. From 2.3.4, the applicants have done a good job describing their impact, indicators and looking at 2.4, the interdisciplinary aspects are made clear. The interdisciplinary aspects of this proposal are strong.

Commentaar referent 2

The major interdisciplinary aspects on the project is the amalgamation of domain expertise in the field of medical science with computer engineering and information technology (Data analytics, AI, ICT technologies etc.). In my professional opinion, if technology like Internet of Things (IoT) and embedded electronics device are considered to be in the scope of the engineering system design implementation of this project then electronics engineering would also be another interdisciplinary aspect the project potentially has. There is a lot of scope do exist in design and implementation of low-power electronics System-on-Chip (Microchips) for IoT based devices for healthcare application which can be custom designed as per application requirement. Even scope exists for Bio-medical sensor design as part of instrumentation engineering.

Commentaar referent 3

The team is very interdisciplinary but I have some concerns on the way the WPs are aligned and the teams really work interdisciplinary.

Weerwoord

@ref2: We agree that there is substantial potential for engineering of specialised low-power electronics. The consortium has several members with relevant expertise in the domain of IOT device engineering (Prof.Kortuem, Dr.Niforatos). The project offers opportunities and concrete design requirements for IoT-based devices that will inform subsequent device engineering after effectiveness of health interventions have been demonstrated. We will focus on device engineering as is required for the overall success of the ID-Buddy system.
We have addressed the concerns of ref3 in our response to comment 4a.

d

To what degree have the different consortium partners been actively involved in drawing up the research plan, and to what degree will they be actively involved in realising it?

Commentaar referent 1

Different consortium partners been actively involved in drawing up the research plan from what has been written

Different consortium partners will be actively involved in realizing it. There is strong use of partners that will be vital to realize the outcomes of the proposed research.

Commentaar referent 2

I believe, the role consortium partners as stated in the proposal (in section 3: Description of work packages) is very systematic and quite convincing.

Commentaar referent 3

It is not clear how they have been involved, but the authors nicely describe their expertise and the way they will be involved in the conduct of the research program.

Weerwoord

@Ref3: A number of the consortium partners have a longstanding collaboration history. We have organized several consortium meetings, and have also discussed the contribution of each partner in one or more separate meetings. Each consortium member contributed to the writing and revision of the project plan.

e

What is your opinion on the feasibility and suitability of the proposed research plan?

Commentaar referent 1

I would have also like to have seen contingency plans if the proposed research did not go as planned, this would have helped my ability to rate the feasibility and suitability as strong. However, as presented, I will rate the feasibility and suitability as good.

All research needs backup plans as often, especially over 6 years, things do not always go as planned.

Commentaar referent 2

In my professional opinion the feasibility and suitability of the proposed research plan largely depends upon following factors. 1) Involvement of End-Users up to a large extent so that more data may be collected (Quality and Quantity of data is the major contributor for AI -based system to work effectively with higher accuracy). 2) Legal and privacy aspects of data to be collected from users. 3) Consortium actual functioning and implementation success and proper effective project management.

Commentaar referent 3

good

Weerwoord

@Ref2: We agree with the reviewer and have these aspects covered in the project, i.e. 1)the field labs (WP2) and prototyping (WP3) ensure co-creation with end-users, 2 the WP-leads are used to work with privacy-sensitive information, and we will be supported and advised by legal and privacy officers at the participating institutions, and 3)efficient management is warranted by the experience in leading large projects from several WP-leads including coordinator Prof.Steegers-Theunissen, the support of a dedicated project-manager, regular meetings and a clear progress monitoring structure.

5 - Final assessment

a

Could you please summarize (point by point) at least three strengths of the grant application, taking into account all assessment criteria?

Commentaar referent 1

1. The interdisciplinary collaboration in the proposed research are strong

2. The societal impact if properly realized of the proposed research is strong

3. The timeliness of the problem being solved at current day and age is strong.

Commentaar referent 2

1. The PI and other consortium members have quite good credentials.
2. The use of Artificial Intelligence (AI) in healthcare sector is quite promising societal and economic impact.
3. NWO requirements of all WP [1-6] are met with very strong planning and detailed functional work plan.

Commentaar referent 3

1. the research is very relevant and it tries to tackle a very important societal problem
2. the consortium consist of a large groups of important stakeholders and partners
3. the scientific team consists of various disciplines.

Weerwoord

We thank the referees for this summary.

b

Could you please summarize (point by point) at least three weaknesses, points for improvement, and / or questions regarding the grant application, taking into account all assessment criteria?

Commentaar referent 1

1. Contingency plans for the different objectives should be addressed.

2. Methodology that will be used at different stages of the timeline that lead towards WP1 to WP6 should be addressed

3. Some details regarding the exact tasks per each Human Capital, how they will work together (when needed) and who exactly and how they will be guided should be made clear.

Commentaar referent 2

1. As it deals with personal healthcare data of users, privacy and legal aspect can be a huge challenge in implementation.
2. The engineering /technology aspect of this project has a limitation as it has only proposed AI for data analytics and a Mobile App (User Interface) doesn't focus much on IoT-based design of smart device or sensors or application specific hardware. This domain has huge potential. Because third party health tracker may not give required data up to the required level of accuracy for effective functioning of AI-driven system. In a nutshell for AI-based system quality and quality (samples/ users) are the key to achieve best results.
3. Does not give much technology plan (Engineering design aspect) the technical know-how to design such proposed system hence difficult to comment of technical feasibility and actual efficacy of system implementation.

Commentaar referent 3

1. The solution is very much technology driven and the lack of potential success is not well described and based on assumptions
2. The WP are well described and refer to each other but they do not convince me that the different disciplinary reseachers will work together as an interdisciplinary team.
3. It is not clear how they will diminish the environmental risks (see societal challenges).

Weerwoord

We hope that our responses to the comments are convincing to tackle these identified weaknesses.

6 - Data management: Feedback

Toelichting

With the data management section, NWO mainly wants to raise awareness about the importance of responsible data management. The section is therefore not included in a committee's decision about whether a proposal should be awarded funding or not. NWO does, however, submit this section to the committee and referees for any advice or feedback. After a proposal has been awarded funding, the applicants should expand the section into a data management plan. For this, applicants can make use of the adv

If you have any suggestions or advice regarding the data management section, please state it here, otherwise type "n/a ".

Commentaar referent 1

Privacy of data gathered will be vital in this proposed research, involvement of young vulnerable families and their data security and privacy will be paramount and should be addressed if this proposal is awarded funding.

Commentaar referent 2

The proposal already mentioned that, they are following industry standard data storage and management system (details available in section.4 of full proposal) and it is adequate hence, I don't have anything more to add to it.

Commentaar referent 3

n/a

Weerwoord

Overall
