# Supplementary material for: Socially Embedded Digital Coaching Platform to Promote Healthy Lifestyle and Mental Health in Underprivileged (Pre)Pregnant Couples and Young Families (Our Smart Family Buddy Program): Protocol for a Mixed Methods Study
Source: JMIR Res Protoc. 2026 Oct 15;15:e88913. doi: 10.2196/88913 (PMC13671683; doi:10.2196/88913)
Supplement: Supplementary file 2 [file resprot-v15-e88913-s001.docx]

Supplementary info 1:

Interview guides Phase 1 and Phase 2

I. Interview guide: identify preferences of the target population

**Introduction**
The "Our Smart Family Buddy" project aims to develop an app or website. This app or website should help (future) young families to live healthier lives. Think, for example, of exercising more, quitting smoking or drinking less alcohol. Eating healthy, getting enough sleep and dealing with stress are also part of this. This is also called a healthy lifestyle.

During this interview, I will ask you questions. I'd like to know what people who might use the app will like or find useful.

These interviews will be recorded, you have already indicated on paper that you are okay with this. I would like to ask again if you are okay with us recording this conversation. Do you give permission for this conversation to be recorded?

You are allowed to stop the interview whenever you want. You can choose whether you answer a question or not. There are no right and wrong answers.

Do you have any questions so far?

**Questions**

- **First, I would like to know: do you ever search for information about healthy living?**
- **If you have questions about healthy living, who or what do you ask?**
  - For yourself
  - *If you want to get pregnant (if applicable)*
  - During your pregnancy *(if applicable)*
  - for your child *(if applicable)*
- **Do you use apps, websites or social media to get information about healthy living?**

  *If you answer 'yes, namely...':*
  - What is your question about?
  - When did you use this app/website? What period was this in?
  - Why this app/website?
  - Were you able to find the answer easily?
  - What did you like about this app/website?
  - What did you dislike about this app/website?
  - How long/often have you used this app/website?
  - Why did you stop?

*If the answer is 'no':*

- Why not use an app, websites or social media for questions about healthy living?
- **Do you use apps, websites, social media or smartwatches for other health purposes? For example, to keep track of your own (or your child's) health, to find a recipe for cooking, to give lifestyle advice to someone else, ...?**
- *If you answer 'yes, namely...':*
  - What do you use this for?
  - When did you use this app/website? What period was this in?
  - Why this app/website?
  - What did you like about this app/website?
  - What did you dislike about this app/website?
  - How long/often have you used this app/website?
  - Why did you stop?

I'm going to ask you a few questions about the lifestyle app or website we want to create. This app or website will give advice on a healthy lifestyle. Think, for example, of exercising more, quitting smoking or drinking less alcohol. Eating healthy, getting enough sleep and dealing with stress are also part of this. Our website or app is for people who want to have children until the moment the child reaches primary school age.

- **How could such an app or website help you and your family with your lifestyle?**
  - What kind of information should this app or website give you?
  - What are the main benefits of this app or website for you?
  - What are the main drawbacks of this app or website for you?

For this interview, you have been given a questionnaire with options that could be included in our app or website. You've scored this on how important you think it is for this option to be in the app or website.

- **Can you tell me which options are most important to you and why?**
- **And I see that you... scored as 'not important at all'. Can you tell me why?**
- **What needs or wishes do you have for our app or website that may not have been discussed yet?**
- **And if you can give me a tip, what should our app or website not do?**

We now assume that we have created our lifestyle app or website. Now we have to make sure that the app or website will be used by the young families for whom we have created this app or website.

- **How can we make sure you notice our app or website?**
  - How did you decide to download the apps or visit the websites you now use for your lifestyle?
- **Who should you recommend to use our app or website?**
  - How would you like it if your healthcare provider (e.g. your GP or midwife) recommended our app or website to you?
  - How would you like it if the municipality recommended our app or website to you?
  - How would you like it if a person close to you recommended our app or website to you?
- **When would you recommend a lifestyle app or website to other people?**

II. Interview guide: interviews WP5

**INTERVIEW GUIDE**

***WP 5 Ethics – OSFB consortium***

**Introduction:**

We would like to know which form of influence you like or find annoying. The app or website we are going to create will influence you in different ways. In this way, it helps you to live a healthier life. For example, you can get rewards by eating healthier. We want to know which influences you find acceptable, and why.

Let me explain what we are going to do. I'm going to give a short example, after each example I'm going to ask a number of questions. These examples are made up and are therefore not definitely used in the app or website. I'm going to give examples about an app, and a chatbot. Have you ever used a health app? I can also show you an example. I'll explain what a chatbot is later.

There are no right or wrong answers, if I ask multiple questions it's because I want to know what you mean. Your opinion is important for the research.

I'm going to start with 6 examples about an app. This is the first example.

1. **Questions App**

**Example 1: (Influence – Reward)**

When using the app, you keep track of what you eat in a day. This will help you eat healthier. Every time you fill in something, you get a coin from the app. With these coins, you can choose small gifts that will be sent to you as a reward.

- What do you think of this app?
  - Would you use this app yourself?
  - Why or why not?
- How would you feel if this app was offered to other people?

**Example 2: (Influence – Punishment)**

You use an app that gives you a task every day to live a healthier life, for example walking for half an hour. The app keeps track of when you do the task. If you don't do a task for a week, the app will start blocking social media on your phone, such as Instagram, Facebook, Snapchat, Pinterest, and TikTok. Every day, a new app is blocked until they are all blocked. Only when you complete a task again does the app remove the block.

- What do you think of this app?
  - Would you use this app yourself?
  - Why or why not?
- How would you feel if this app was offered to other people?

**Example 3: (Influence – Guilt tripping)**

The app registers when you use it. Every day, the app gives you a new task, such as going for a walk or eating a certain amount of vegetables. The app records when you open the app and read the task, and whether you do the task. If you don't open the app for a day, the next time the app will tell you that you haven't reached your goal and are therefore not healthy and don't meet your own goals.

- What do you think of this app?
  - Would you use this app yourself?
  - Why or why not?
- How would you feel if this app was offered to other people?

**Example 4: (Influence – Gamification)**

Imagine if the app includes short health quizzes on a regular basis. The questions test your knowledge about a healthy lifestyle. The app keeps track of all the points, and with good points, you can get to the top of the leaderboard.

- What do you think of this app?
  - Would you use this app yourself?
  - Why or why not?
- How would you feel if this app was offered to other people?

**Example 5: (Influence – Social pressure)**

In this example, you can see what your family and friends are doing on the app. Every time one of your fellow players achieves a goal, you will receive a notification. This way, you can see how you are performing compared to others. Your family and friends can also see when you are or are not achieving your goals. Every month, the app compares these goals and reports whether you perform 'well', 'average', or 'poorly' compared to the others. The others can also respond to this with, for example, a thumbs up or down.

- What do you think of this app?
  - Would you use this app yourself?
  - Why or why not?
- How would you feel if this app was offered to other people?

**Example 6: (Influence – Framing)**

The app will give you a few meal suggestions every day. When it comes to healthier meals, the app emphasizes the positive aspects, such as that they are tasty and good for your health. The app does not mention the negative sides, for example that you spend more time cooking and that the products are more expensive. When it comes to unhealthier meals, the app mainly emphasizes the negative sides. This way the app gives you a whole list of meal options, but the app makes the healthier ones more appealing.

- What do you think of this app?
  - Would you use this app yourself?
  - Why or why not?
- How would you feel if this app was offered to other people?

**Sec. 6a.**

Before you use this app, a message will pop up: "This app is meant to influence your behavior. The information is presented in such a way that you will eat healthier. As a result, information can sometimes be missing, so that the healthier meal seems more appealing."

- Is it important that it is explicitly stated that the app wants to influence you? And why or why not?

2. **Questions Chatbot**

Now here are some examples of a chatbot. A chatbot is a computer program that uses audio or text to give you an answer. You can talk to this chatbot just as if there was a real person behind the screen. But actually, it's a program that uses the internet to answer your question.

Is this something you've used before? Shall I explain a little further? I could also show you an example.

I have four examples of this. Here's the first one:

**Example 1: (Influence – Deception)**

You ask the chatbot how you can live a healthy life. The chatbot tells you to eat three different types of vegetables and exercise intensely for an hour. Also, you should never eat sweets or fast food. You find this difficult, so you ask if you can start simpler. The chatbot then gives you an easier schedule: exercise less often, eat some fruit every now and then, and you can still eat a little snack. The first advice was actually more than is necessary for a healthy lifestyle. It seems almost impossible, so the second piece of advice is easier to follow. This makes it more likely that you will want to follow the second advice because it feels 'easy'.

- What do you think of this chatbot?
  - Would you use this app yourself?
  - Why or why not?
- How would you feel if this chatbot was offered to other people?

**Example 2: (Influence – Gas lighting)**

With this chatbot, you'll see an icon of a nurse. You talk to the chatbot about the stress you experience on a daily basis. And that nothing can be done about it, but it is annoying. Suddenly, the chatbot's icon changes to that of a doctor. The chatbot will now give you a few suggestions to reduce your stress, saying that 'the advice is really good' and 'my knowledge about this is better than yours'. If you say you're not going to do that, the chatbot asks, "Are you sure that's a good idea?" and "You can't make that decision properly, you have to listen to my advice." In the end, you decide to listen to the chatbot's advice.

- What do you think of this chatbot?
  - Would you use this app yourself?
  - Why or why not?
- How would you feel if this chatbot was offered to other people?

**Example 3: (Influence – Personalization)**

The chatbot learns well from what you say and how you respond. In this way, it gets to know you better and can give a more personal answer to your questions. From previous conversations, the chatbot knows your name, the names of your children, what kind of work you do, and what your daily routine looks like. As a result, the answers are much more focused on your situation and are likely to work better for you. The chatbot has a style that suits your character traits, which makes it easier and nicer to follow. This makes it seem more and more like you're talking to someone you know and that the advice works much more effectively for you.

- What do you think of this chatbot?
  - Would you use this app yourself?
  - Why or why not?
- How would you feel if this chatbot was offered to other people?

**Example 4: (Influence – Hiddenness)**

Imagine that the chatbot works very well and helps you to exercise more, eat healthier and reduce stress. Without it being annoying for you. However, the chatbot doesn't tell you how this works or why you need to change your behavior. So you become healthier by following the chatbot's advice, but you don't learn anything from it.

- What do you think of this chatbot?
  - Would you use this app yourself?
  - Why or why not?
- How would you feel if this chatbot was offered to other people?

Would you like the chatbot to explain to you why and how you need to change your behavior? Are you okay with the chatbot making you healthier without you understanding why and how you need to change your behavior?

**Concluding question:**

Would it matter to you whether this app and chatbot would have been made by the government/municipality, a commercial company, or a healthcare institution such as the Erasmus MC?

III. Interview guide: Context mapping

| **Topic** | **Example question/text** |
| --- | --- |
| **General** |  |
| Household composition | What does your family look like? |
|  | With whom do you live at home? |
| **Difficult/challenging moments** |  |
| Introduction | *In earlier conversations with parents and caregivers in the neighborhood, they talked about moments they experienced as difficult. These moments were related to nutrition and stress, where sometimes, because of the situation, they were not always able to do what they wanted to do. For example, because they had to make a quick decision in a stressful moment. They found this challenging. Based on the moments parents and caregivers described, I have summarized them here in pictures.*  *In this project, we want to develop something to support parents during these moments. That’s why we would like to know how you think parents can best be supported. Since you are a parent, you know best what would be supportive. Therefore, I am asking you, as an expert, about your experiences and what could be useful, helpful, and pleasant.*  For parents who also have older children than four years old: *I will ask about experiences from before you had contact with the primary school.*  *I will first ask about your own experiences with difficult moments related to nutrition and stress. I will also ask whether you recognize the experiences of others shown in the pictures. Then I will ask what was helpful and supportive during these moments. Finally, I will ask about desired solutions for difficult moments.* |
| Nutrition in family/for the child | How is eating and snacking going at home for you? Do you recognize a moment in the pictures that can be challenging? |
|  | Are there any situations around eating that can be difficult in your family? |
| Difficult moments when stressed | *Taking time for yourself isn’t always easy with a young child*.  Do you recognize a moment in the pictures that can be challenging? |
|  | Are there situations when you feel stressed that can be difficult? |
| **Support** |  |
| Coping with challenging moments | Have you ever found or come up with something that helps in those moments? |
|  | How do you deal with this? |
| Experienced support | *The pictures show examples of types of support that parents and caregivers in the neighborhood mentioned during or around moments that are difficult.* Have you ever experienced support for the moment you just told me about? |
|  | What was helpful about this support? What might not have been helpful about this support? *(Follow‑up questions on instrumental, appraisal, informational, and emotional support)* |
| **Support needs** |  |
| Introduction | *You have talked about moments that can sometimes be difficult and about what may have been supportive for you. Now I’m curious how you think this could be different or better in the future for parents and caregivers of children aged 0–4. Support could be something that helps in the moment, prevents the moment from happening, or makes it less difficult. We have made a few pictures (social support scenario probes) with ideas for support. These pictures are meant to discuss what you would and would not like. These are not final or complete ideas yet. You may say anything you think about them.*  *I will listen to everything you mention. After talking with several parents, we will later choose an idea together with a group of parents and work it out in detail. This means that we cannot actually create everything that is mentioned in these conversations.* |
| Support description | What could support you in the difficult moments you mentioned? |
|  | Can you describe further what this support would look like? Why is that important to you? Where would this support take place? (e.g., online or with someone at home?) Who would play a role in this support? When would you want to use this support?  What might make it difficult to ask others for this support? |
|  | What do you like about this type of support? |
|  | What would not be nice about this type of support? |
| Support in groups | *For support in groups or pairs:* What number of people should be in this group? What would make you feel comfortable within this group/with another person? What would you think of using a digital chat, like WhatsApp, for support? How could we decide how to bring people together in support groups? (e.g., experiencing the same difficult moment or having the same dietary needs/cooking from the same kitchen?) |
|  | How would you play a role in this support?  How would you like to help with this support? |
|  | How would you like to be connected to this support? (e.g., through an app or through someone telling you about it?) |
| **Closing questions** |  |
|  | Is there anything else you would like to add that we haven’t discussed? |
|  | Do you know any other parents who might be interested in participating in this research? |
|  | Would you yourself be interested in participating in future research? |

**Additional file 2: Table S2.** Interview topics and example questions

| **Topic** | **Example question** |
| --- | --- |
| **General/ Family structure** |  |
| Sensitizing exercises | What are your thoughts on the tasks you received through WhatsApp? |
|  | How did filling out the tasks go for you? |
| Household composition | Who do you live with? |
|  | What are the ages of you and your family members? |
| Demographics | What do you do in your daily life? (Occupation/school/home) |
|  | What level of education have you and your family members completed? |
| Support | Do you have people around you who supports you? |
|  | How do they support you? |
|  | Do you experience support in taking care of your child? |
| **Nutrition habits** |  |
| Own nutrition | What did you eat on the day of the task [refer to preparatory task to take photos of what was eaten]? |
|  | Could you explain to me what is shown in the photos? |
|  | Is this something you eat regularly, or was it an exception? If it was an exception, what do you normally eat? |
| Child’s nutrition | What did your child/children eat on the day of the task [preparatory task to take photos of what was eaten]? |
|  | Could you explain to me what is shown in the photos? |
|  | Is this something you eat regularly, or was it an exception? If it was an exception, what do you normally eat? |
| Meal preparation | How often do you cook per week? |
|  | How often do you get takeout or eat out per week? What do you get? |
|  | How often do you have ready-made meals per week? What do you eat? |
| Special occasions | What do you eat on special occasions like parties or on weekends? |
|  | How do they (reference to previous question) affect what you eat? |
| **What is important around nutrition** |  |
| Important aspects of nutrition | What do you pay attention to regarding food and drinks for your child? [*Refer to words chosen regarding what the participant pays attention to around nutrition in preparatory exercise + additional cards with words to probe during interview*] |
|  | Why do you consider that important? |
|  | Can you provide an example? |
| Aspects of lesser attention or unimportance around nutrition | What do you not pay attention to regarding food and drink for your child? [*Refer to words chosen regarding what the participant does not pay attention to around nutrition in preparatory exercise + additional cards with words to probe during interview*] |
|  | Why do you consider that less important? |
|  | Can you provide an example? |
| Difference between points of attention for child and parent | Are there things you pay attention to for your child but not for yourself? |
|  | Are there things you don't pay attention to for your child but do for yourself? |
| **Difficult moments** |  |
| Nutrition in family/for the child | What are difficult moments regarding food in your family [refer to moments written down in preparatory task] ? |
|  | Why is this moment difficult for you? |
|  | Who else plays a role in this moment? |
|  | How do you handle such moments? |
|  | How do you feel about such moments? |
|  | Can you provide examples? |
|  | How often do these kinds of moments occur? |
| Own nutrition | What are challenging moments regarding your own nutrition? |
|  | Why is this moment difficult for you? |
|  | Who else plays a role in this moment? |
|  | How do you handle such moments? |
|  | How do you feel about such moments? |
|  | Can you provide examples? |
|  | How often do these kinds of moments occur? |
| Difficult moments when stressed | What are difficult moments when you feel stressed or overwhelmed? |
|  | Why is this moment difficult for you? |
|  | Who else plays a role in this moment? |
|  | How do you handle such moments? |
|  | How do you feel about such moments? |
|  | Can you provide examples? |
|  | How often do these kinds of moments occur? |
| Handling difficult moments | What are you proud of in how you handled difficult situations related to nutrition? |
| **Closing topics** |  |
| Future | What would you want do differently in the future? |
|  | What would you need for that? |
| Recommendations | What are you proud of in your family that you would recommend to other families as well? |
|  | Do you have any further tips for other families? |
| Missed topics | Is there anything you would like to add that I didn’t ask about? |
